# Bortezomib: killing two birds with one stone in gastrointestinal stromal tumors

**DOI:** 10.18632/oncotarget.103

**Published:** 2010-05-01

**Authors:** Stefan Duensing, Anette Duensing

**Affiliations:** ^1^ Cancer Virology Program, University of Pittsburgh Cancer Institute, Hillman Cancer Center, Pittsburgh, PA, USA; ^2^ Department of Microbiology and Molecular Genetics, University of Pittsburgh School of Medicine, Pittsburgh, PA, USA; ^3^ Department of Pathology, University of Pittsburgh School of Medicine, Pittsburgh, PA, USA

Gastrointestinal stromal tumors (GIST) are the most common mesenchymal tumors of the gastrointestinal tract and are caused by activating mutations of the *KIT* or *PDGFRA* receptor tyrosine kinase genes [1-3]. This causative relationship between single gene mutation and tumor formation was key for the extremely successful introduction of the small molecule inhibitor imatinib mesylate (Gleevec) in the treatment of this hitherto deadly disease. Despite this success story with greater than 85% of patients with metastatic disease benefiting from imatinib therapy [4], there are a number of problems. Remissions are often incomplete and, most importantly, the majority of patients develop resistance to imatinib during the course of treatment [5]. The most common mechanisms of imatinib-resistance are additional, secondary mutations of *KIT* or *PDFGRA* that affect the conformation of the kinase domain and therefore make imatinib unable to bind while the oncogenic kinase activity is retained [6]. Unfortunately, only a subset of imatinib-resistant mutated kinases are sensitive to sunitinib, a multi-targeted kinase inhibitor approved for the treatment of imatinib-resistant GISTs [7, 8]. The development of alternative approaches to treat patients with GIST that failed first and second line therapies is hence imperative.

The prevailing mechanism of GIST cell eradication is through apoptotic cell death, although recent studies have shown that a fraction of imatinib-treated tumor cells do not die but instead enter quiescence [9]. Since such cells can serve as seeds for relapse and potentially refractory disease, it is desirable to push as many tumor cells as possible towards apoptosis. Studies analyzing the dynamics of GIST cell apoptosis following imatinib treatment led to the puzzling observation that kinase inhibition was a rapid effect that occurred within minutes whereas the onset of apoptosis took considerably longer with maximum cell death after approximately three days. While these results suggest that kinase inhibition plays a pivotal role in inducing GIST cell apoptosis, they left unanswered what may happen in the lag period between kinase shut-down and apoptosis. A study published in 2007 by Liu and co-workers provided insights into this question by showing that imatinib-treated GIST cells developed a massive upregulation of a histone protein of the H2A family, histone H2AX [10]. H2AX is well known for its role in the cellular response to DNA double-stranded breaks [11]. Liu and co-workers could demonstrate that H2AX was a potent inducer of apoptosis in GIST cells and that this novel function was not primarily associated with its role as nucleosomal factor in the DNA damage response. Instead, induction of apoptosis was a novel, non-nucleosomal function of this protein since the majority of it was found to be in soluble subcellular fractions. Mechanistically, free H2AX may non-specifically bind to DNA and/or chromatin thereby blocking transcription and causing cell death. Importantly, Liu and co-workers made the observation that H2AX was regulated by the ubiquitin-proteasome machinery in GIST cells and that proteasome inhibition led to increased levels of H2AX. These findings laid the groundwork for the recent study by Bauer and co-workers [12] who asked the question whether it would be possible to trigger GIST cell apoptosis with the FDA-approved proteasome inhibitor bortezomib (Velcade). Bortezomib has previously been used mostly for the treatment of multiple myeloma, a hematologic malignancy that affects plasma cells [13]. Bauer et al. showed that bortezomib causes cell death not only in imatinib-sensitive GIST cells but also in imatinib-resistant GIST cells. These results provided compelling evidence that the induction of apoptosis is still possible despite the presence of resistance mutations when the proper downstream mechanisms were targeted. Obviously these results prompted an in depth analysis of the mechanisms underlying the response to bortezomib. As expected from previous studies, bortezomib-treated cells showed a significant increase of the levels of soluble H2AX. Surprisingly, this was not the only mechanism leading to GIST cell apoptosis as Bauer et al. also found that bortezomib-treated cells showed an almost complete loss of the KIT kinase protein itself. Further follow-up studies revealed that this downregulation of KIT expression was caused by a massive inhibition of transcription similar to the transcriptional shut-down inducible with the RNA polymerase II inhibitor a-amanitin. Although one possible explanation for this finding was that the increase of H2AX was causing the decreased transcription of KIT, depletion of H2AX only incompletely rescued cells from bortezomib suggesting additional effects to be involved and no downregulation of KIT protein is usually seen after imatinib treatment. Interestingly, inhibition of the NF-ĸB signaling cascade, a key mechanism of action of bortezomib reported in multiple myeloma [14], seemed not to play a major role in bortezomib-treated GIST cells. Collectively, these results suggest that bortezomib has a dual negative effect on GIST cell viability via upregulation of the pro-apoptotic histone H2AX and downregulation of the oncogenic KIT kinase.

A number of important questions arise from these findings. First, the source of soluble, pro-apoptotic H2AX in GIST cells remains to be determined. It is not clear whether the increase is triggered by newly synthesized H2AX or whether the protein has been evicted from chromatin. Second, the mechanisms that normally limit H2AX levels in human cells remain to be determined. Given the fact that H2AX is polyubiquitylated, the identification of an E3 ubiquitin ligase should yield important insight in its regulation. Related to this problem is the question how KIT interferes with the H2AX degradation machinery to keep H2AX low. Although the pro-apoptotic activities of H2AX are carried out by soluble protein, its constitutive downregulation may cause a global depletion of the protein in cellular nucleosomes and hence impair the response to DNA damage. It will be important to test whether re-expression of H2AX can restore GIST cell sensitivity to DNA damaging agents such as chemotherapeutic drugs or radiotherapy, which could be exploited for combination therapies.

The dramatic loss of KIT protein expression following bortezomib treatment likewise raises several questions. It is currently unclear what the precise mechanisms of the transcriptional shut-down in bortezomib-treated GIST cells may be. The most straightforward explanation would be that inhibition of the proteasome leads to an accumulation of negative transcriptional regulators. This effect is unlikely to be sequence-specific but rather targeting the global transcriptional machinery, such as the loss of initiating and elongating RNA polymerase II, but not total RNA polymerase II protein, an effect that is seen in bortezomib-treated GIST cells. A disruption of the proper proteolytic turnover of transcription factors or components of the transcriptional machinery is another possibility that needs to be taken into consideration. It has been shown that the ubiquitin-proteasome machinery is critically involved in the regulation of transcription initiation and elongation [15]. Another critical question is why GIST cells are so sensitive to the loss of ongoing KIT transcription. We (unpublished results) and others [16] have shown that mutant KIT protein is less stable compared to wildtype protein, indicating that its constant production is necessary to provide constant oncogenic stimulation. A translational aspect of these results would be to test whether transcriptional inhibitors can be used to induce GIST cell death. Whether bortezomib also affects other pathways such as chaperone proteins to downregulate KIT protein expression remains to be determined.

**Fig. 1:**
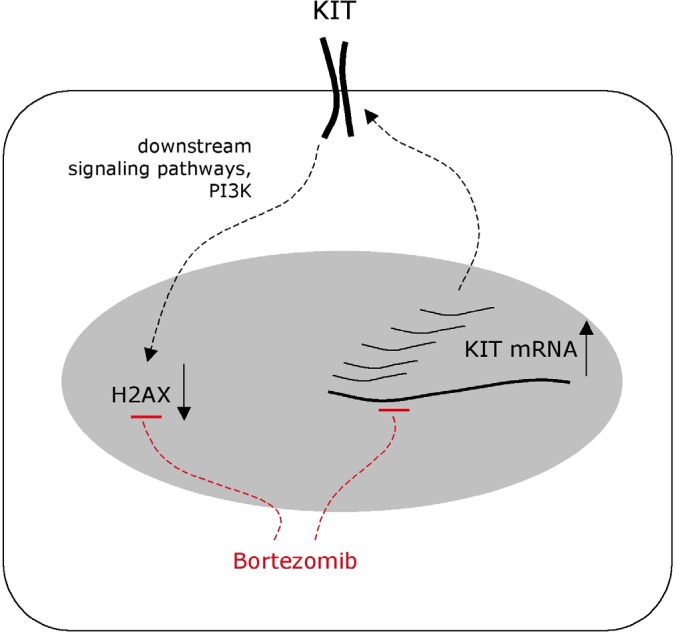
Bortezomib reverses two aspects of GIST cell biology. GIST cells rely on a high rate of ongoing KIT transcription in order to maintain continuous expression of the less stable mutant protein and hence oncogenic signaling. Previous results have shown that one function of oncogenic KIT is to downregulate expression of the pro-apoptotic histone H2A family member H2AX through protein degradation. Treatment of GIST cells with bortezomib was found to increase H2AX levels, which can lead to soluble non-nucleosomal H2AX and enhanced cell death. At the same time, bortezomib was found to trigger an almost complete loss of KIT protein expression involving a transcriptional shut-down. Soluble H2AX itself can block ongoing gene transcription, so it is possible that this activity contributes to the downregulation of KIT transcription in bortezomib-treated cells. It is also possible that downregulation of other mRNAs than KIT plays a role in this process since a general inhibition of RNA polymerase II-mediated gene transcription was observed. Note: The precise intracellular site and mechanism of H2AX proteasomal degradation remains to be determined.

Even without considering other downstream effectors of oncogenic KIT, the signaling pathways that control H2AX and KIT expression in GIST cells are likely to be extremely target-rich. There are hence realistic expectations that more specific approaches can be developed for future interventions against GISTs with an aim towards cure.
